# Spatial variations and determinants of receiving cash and food from the productive safety net program among households in Ethiopia: spatial clustering and multilevel analyses

**DOI:** 10.3389/fpubh.2024.1392111

**Published:** 2024-07-15

**Authors:** Bewuketu Terefe, Begosew Muluneh, Wullo Sisay Seretew, Bisrat Misganaw Geremew

**Affiliations:** ^1^Department of Community Health Nursing, School of Nursing, College of Medicine and Health Sciences, University of Gondar, Gondar, Ethiopia; ^2^Department of Epidemiology and Biostatistics, Institute of Public Health, College of Medicine and Health Sciences, University of Gondar, Gondar, Ethiopia

**Keywords:** cash and food reception, spatial variation, households, productive safety net program, multilevel analysis, Ethiopia

## Abstract

**Background:**

There is a global struggle with food insecurity and undernutrition among women, and Ethiopia has been particularly impacted by these issues. To address this challenge, Ethiopia has implemented a cash and food safety net program over many years. However, there is limited information available regarding the program’s factors and spatial distributions, with no recent national evidence from Ethiopia. Consequently, the objective of this study is to investigate the spatial clustering and determinants of the Productive Safety Net Program (PSNP) in Ethiopia.

**Method:**

This study utilized data from the Ethiopian Demographic and Health Survey. The sample included 8,570 weighted households. Given the hierarchical nature of the data, a multilevel logistic regression model was employed to identify factors influencing the outcome variable. Geographical clusters of individuals receiving assistance from the PSNP were examined using SaTScan software and the Bernoulli model, along with the Kulldorff methods. The nationwide distribution of the program beneficiaries was visualized using ArcGIS version 10.8. Variables were considered statistically significant if their *p*-value was <0.05.

**Results:**

The overall coverage of the PSNP was 13.54% [95% confidence interval (CI): 12.84–14.29] among households in Ethiopia. The study revealed that people from richer households adjusted odds ratio [AOR = 0.46 (95% CI: (0.33, 0.64))], those from the richest households [AOR = 0.26 (95% CI:(0.17,0.41))], and those with educated household heads [AOR = 0.45 (95% CI:(0.28, 0.71))] have a lower likelihood of utilizing the PSNP compared to their counterparts. Conversely, a unit increase in household heads’ age [AOR = 1.02 (95% CI:(1.01, 1.02))] and family size [AOR = 1.05 (95% CI:1.021.10)] showed a higher likelihood of joining the PSNP, respectively. Household heads who have joined community health insurance [AOR = 3.21 (95% CI:(2.58, 4.01))] had significantly higher odds of being included in the PSNP than their counterparts. Heads who belong to a community with a high poverty level [AOR = 2.68 (95% CI:(1.51, 4.79))] and community health insurance [AOR = 2.49 (95% CI:(1.51, 4.11))] showed more inclination to utilize the PSNP compared to their counterparts.

**Conclusion:**

PSNP was judged to have a low implementation status based on the findings gathered regarding it. We found factors such as age, sex, region, wealth, education, family size, regions, and health insurance to be statistically significant. Therefore, encouraging women empowerment, community-based awareness creation, and coordination with regional states is advisable.

## 1 Introduction

One of the major developmental challenges faced by Africa in the 21st century is insufficient access to food (1). Achieving food security remains a significant and evolving struggle in developing countries. Food insecurity adversely affects people’s health, education, work capacity, human rights, and equality. Vulnerability to the effects of food insecurity is particularly pronounced among women and children due to their limited access to and control over resources (2, 3). The implementation of social protection programs aimed at reducing poverty has become widespread (4). In Africa, the number of social protection programs has tripled in the last 15 years, with every African country currently operating at least one such program (5, 6). However, reports indicate that, by 2023, 660 million people could still face hunger, and between 720 and 811 million people faced severe hunger in 2020 due to the COVID-19 pandemic (7, 8). These figures demonstrate that the aim of the 2030 Sustainable Development Goals to eradicate hunger may not fully be achieved. The implementation of these programs has been accompanied by discussions regarding various technical aspects, including targeting, payment levels and frequency, program duration, and whether benefits should be tied to specific behaviors. One crucial design consideration is the payment modality: cash or in-kind. It has been reported that approximately 130 low- and middle-income countries have at least one cash transfer program, while the use of in-kind payments, such as food, in social programs remains prevalent (9, 10).

A significant portion of the food aid provided to Ethiopia has been distributed as free aid (11). Achieving food security at the household level is widely recognized as one of the most effective strategies for protecting all family members from food insecurity and reducing poverty because it diversifies the economic bases of the poor (12, 13). According to a 2012 World Bank report, social safety net programs in African nations have expanded rapidly over the past two decades to combat poverty, food insecurity, and the vulnerability of low-income households (14, 15). One of the largest social safety net programs in sub-Saharan Africa is Ethiopia’s Productive Safety Net Program (PSNP), which has been in place since 2005. Along with addressing income disparities, the PSNP aims to regulate household consumption, promote investment in human capital and productive assets, protect household assets, and empower individuals living in poverty to take control of their circumstances (15, 16). However, the available data do not fully support the program’s effectiveness in reducing food insecurity, promoting health, and improving nutritional status. Existing research suggests that safety net programs have enhanced food security, livestock ownership, healthcare service utilization, dietary diversity, healthcare expenditure, nutritional status, and shock resilience (17, 18).

Consequently, it is crucial to critically evaluate the impact of food aid on other development programs (19, 20), examine food subsidies (21), assess potential dependency syndromes in recipient nations and households, and evaluate the efficiency of food aid allocation. Although there is a considerable body of literature on the effects of dietary assistance, studies investigating its impact on the PSNP are currently limited. This study aims to address this gap by analyzing the spatial distribution and allocation of food aid across households, taking into consideration various individual and community-level sociodemographic variables. Investigating how the PSNP affects Ethiopian households’ access to food will provide valuable insights for program implementers, policymakers, and other stakeholders. It is crucial for policymakers and implementers to study the PSNP using advanced models that incorporate community and contextual factors, along with spatial variations. This approach helps in identifying and making decisions regarding the allocation of the PSNP benefits to Ethiopian households without revealing confidential information. Spatial studies have the potential to provide policymakers with valuable insights by visually representing the distribution of the PSNP benefits and highlighting areas that have either received or not received these benefits. This spatial analysis enables effective and efficient decision-making, which, in turn, minimizes delays and resource wastage in the implementation of the program. To the best of our knowledge, no data have been recorded regarding the implementation status of the PSNP in Ethiopia. This study will provide a unique perspective on the PSNP and offer insightful policy recommendations to optimize the program’s impact, which will deepen our understanding of the issue. Therefore, the objective of this study is to investigate the spatial clustering and determinants of the PSNP among households in Ethiopia.

## 2 Materials and methods

### 2.1 Study design, period, and setting

A community-based cross-sectional study was conducted in Ethiopia from March to June 2019 (22). Ethiopia, located in East Africa’s Horn of Africa (3° - 14° N and 330° - 48° E), consists of nine regional states (Afar; Amhara; Benishangul-Gumuz; Gambella; Harari; Oromia; Somali; Southern Nations, Nationalities, and People’s Region (SNNP); and Tigray), along with two city administrations (Addis Ababa and Dire Dawa). It encompasses 16,253 kebeles, 817 districts, and 68 zones, which are the lowest administrative units within the country. With a population exceeding 110 million, Ethiopia is home to a 1:1 sex ratio to the general population, with 39.81% of the population being under the age of 14 years. The country also experiences a death rate of 5.8% per 1,000 people, an urbanization rate of 22.2%, and a high prevalence of major infectious diseases (23, 24). This study utilized the most recent Ethiopian Demographic and Health Survey (EDHS 2019) to examine the geographic distribution and factors influencing the receipt of cash or food from the PSNP in Ethiopia. The Demographic and Health Survey (DHS) collects data on various topics related to households, women’s health and well-being, and interpersonal violence. The survey is nationally representative and includes a weighted sample of 8,666 households. We combined data from households surveyed in the EDHS 2019 to conduct the analysis on the receipt of money or food from the PSNP and explore its potential geographic variations.

### 2.2 Study population and sampling techniques

The EDHS sample was divided into two groups before selection. The regions were stratified into urban and rural areas, resulting in 21 sampling strata. Within each stratum, enumeration areas (EAs) were independently selected in a two-stage process. To achieve implicit stratification and proportional allocation at lower administrative levels, the sampling frame within each stratum was classified based on various administrative units. The first stage of sampling involved selecting 305 EAs (93 in urban areas and 212 in rural areas), with a probability proportional to the size of the EA. Household listing operations were conducted in all selected EAs between January and April 2019, and the resulting household lists served as the sampling frame for the subsequent stage of household selection.

In the 2019 EDHS, there were some EAs with a large number of households, exceeding 300. These significant EAs were split to alleviate the burden of household listing operations. The selection probability was proportional to the segment size, and only one segment was chosen for the survey. Each 2019 EDHS cluster represented either an EA or a component of an EA, and the household listing operations were performed exclusively in the selected segment. In the second round of selection, a fixed number of 30 households per cluster were chosen using equal likelihood systematic selection. Further details on the sampling procedures can be found in the EDHS report (22).

### 2.3 Data quality, collection tools, and procedure

Several measures were implemented to ensure the quality of the DHS data. These measures encompassed training programs for data collectors, supervisors, and field editors; continuous supervision throughout the data collection process; the utilization of standardized and translated questionnaires in international, national, and local languages specific to each country; and the engagement of data processing specialists for efficient data entry and management. A concerted effort was made to address any potential systematic bias. To guarantee data accuracy, a pre-test was conducted before the actual data collection took place. A debriefing session with the pre-test fieldworkers was held to collect feedback, during which any necessary modifications to the questionnaires were mentioned. Further comprehensive guidance on the data collection process can be found in the DHS documentation. The DHS carries out data collection every 5 years, employing trained professional data collectors who are representative of the national population and possess a deep understanding of the unique demographic and health challenges faced by each country. The surveys conducted encompass various aspects, including households, women, men, biomarkers, and health institutions. Once the data were obtained from the DHS, they were subjected to proper data management practices. These practices involved the integration of female and male subjects’ data, the implementation of appropriate techniques to handle missing observations that occur completely at random, and the execution of recoding and variable recategorization as needed. These meticulous steps were taken to ensure the integrity and reliability of the collected data (25).

### 2.4 Data management and model formulation processes

After obtaining online permission and providing a description of the study’s purpose, access to the official database of the DHS program at www.measuredhs.com was granted. The household dataset obtained from the EDHS was utilized to extract the outcome variable along with significant predictors. Data extraction was performed using STATA version 17, and Microsoft Excel was used for geographical analysis and community-level parameter calculations. A bivariable two-level mixed-effect logistic regression analysis was conducted to compute odds ratios with 95% confidence intervals (CIs), aiming to identify independent factors of presumed negative sentiment polarity (PNSP). These identified variables were then included in the final model of the multivariable two-level mixed-effect logistic regression analysis. Statistical significance was determined as *p*-values below 0.05. The fixed and random effects were examined to assess individual and cluster variability. In addition, the study provided comprehensive details on community characteristics and individual personalities. The investigation presented four models: Model Zero (null model) with only the outcome variable, Model I with only individual-level variables, Model II with only community-level variables, and Model III with both individual- and community-level variables. Socioeconomic and demographic information was also collected from each household.

Furthermore, this model served as a reference for adopting a multilevel statistical framework and comparing it with conventional logistic regression. Various statistical measures including proportional change of variance (PCV), log-likelihood ratio test (LLR), median odds ratio (MOR), intraclass correlation coefficient (ICC), and Akaike information criterion (AIC) were used to analyze the null model. The measure of variation was evaluated using the MOR, which is the median value of the odds ratio between the area at the lowest risk and the area at the highest risk when two clusters are randomly selected. MOR = e0.95√VA or MOR = exp. [√ (2 × VA) × 0.6745], where VA is the area level variance (26, 27). The PCV reveals the variation in PSNP utilization among households explained by factors. The PCV was calculated as= $\frac{\text{Vnull}-VA}{\text{Vnull}}$*100, where Vnull is the initial model’s variance and VA is the model’s variance with additional terms. Moreover, the ICC, a measurement of the variation in PSNP utilization between clusters, was computed as ICC = VA÷VA + 3.29 ∗100%, where VA = area/cluster level variance (26, 27).

### 2.5 Spatial analysis

To prepare the data for spatial analysis, we cross-tabulated the weighted frequency of the dependent variables and the cluster numbers to obtain the case-to-total proportion ratio (ArcGIS). The input was then combined with the outcomes. To determine whether the data pattern was concentrated, distributed, or random over the study area, we first cleaned and removed data with zero latitude/longitude coordinates. The spatial analysis was conducted using ArcGIS version 10.7 software, which is a widely used software tool for analyzing geographic data. ArcGIS provided a comprehensive set of spatial analysis capabilities, allowing for the exploration and visualization of spatial patterns, relationships, and trends in the data. In addition, SaTScan version 10.1 software was utilized for conducting specific SaTScan analyses. SaTScan is a specialized software program designed for spatial and temporal cluster detection in epidemiological studies. It enabled the identification of statistically significant local clusters with high rates and low rates of events.

### 2.6 Spatial autocorrelation analysis

Spatial autocorrelation analysis was conducted to visualize the spatial patterns of PNSP among households in Ethiopia. The Global Moran’s I statistic was employed to determine whether the distribution of uptake PNSP among households in Ethiopia exhibited a dispersed, clustered, or random pattern (28, 29). Moran’s I statistic produces a single output value ranging from −1 to +1, where values close to −1 indicate a dispersed pattern, values close to +1 indicate a clustered pattern, and a value of 0 suggests complete randomness. A statistically significant Moran’s I value (*p* < 0.05) indicates the presence of geographical clustering (28, 30). This statistic is based on Tobler’s first law of geography, which states that things that are geographically closer are more closely related than those that are farther apart (31). To assess the global spatial autocorrelation, we employed the inverse-distance conceptualization to establish spatial relationships and utilized the Euclidean distance method with raw standardization to mitigate any biased distribution of spatial features. The Global Moran’s I statistic was utilized to provide a comprehensive measure of the spatial autocorrelation in the dataset. However, it should be noted that the extent of spatial autocorrelation could vary considerably across different geographic areas.

### 2.7 Hot spot analysis (Getis-Ord Gi* statistic)

The Gettis-OrdGi* data were created to determine how the geographic autocorrelation of PNSP changes across Ethiopia. The statistical importance of the clustering of the target variable over the study region at various significance levels was immediately identified using a hotspot analysis, which produces a Z-score and a *p*-value. A statistical output with a high GI* denotes a “hotspot” (high PNSP reception or utilization), whereas a statistical output with a low GI* denotes a “cold spot” (low PNSP reception or utilization). Therefore, we employed the local indicators of spatial autocorrelation (LISA) statistics to detect and visualize the variations in spatial autocorrelation across different regions in Ethiopia (28, 29). The LISA statistics provided disaggregated estimates at different locations.

### 2.8 Spatial interpolation

The ordinary Kriging and Empirical Bayesian Kriging methods were utilized in this research because they consider spatial autocorrelation and statistically optimize the weight. The PNSP utilization practices in unobserved regions of the nation were predicted using the standard Kriging spatial interpolation technique (32). This method helped to evaluate the strength of clustering across different areas.

### 2.9 SaTscan statistics

A spatial scan (SaTScan) statistical analysis was conducted using the SaTScan method to identify important primary and secondary clusters of the PSNP based on the Bernoulli distribution (33). In this analysis, a circular scanning window was employed, which moved across the study areas. Household members who utilized the PSNP were considered as cases, while those who did not utilize it were considered as controls to fit the Bernoulli model. The scanning window with the maximum likelihood represented the cluster with the highest likelihood of significant performance (33). The distribution and statistical significance of clusters were explored using Monte Carlo replication with more than 999 replications, ensuring sufficient power in the cluster identification process. Green-colored dots indicated a high proportion of the PSNP, while yellow- and red-colored dots indicated a low proportion of the PSNP. The likelihood ratio (LR) test statistic and its corresponding *p*-value were utilized to determine whether the observed number of households undergoing the PSNP implementation within a potential cluster was significantly higher or lower than expected. For each identified cluster, the LLR test statistic, along with its associated *p*-value, relative risk (RR), location radius, population, and number of cases, was reported.

### 2.10 Dependent and independent variables

The dependent variable in this study was whether the household received cash or food from the PSNP. The outcome variable was categorical, with “Yes” assigned a value of 1 and “No” assigned a value of 0. The independent variables included the sex and age of the household head, the level of education, residence, wealth status, family size, presence of children under the age of five, and health insurance. In terms of community-level factors, the analysis considered the following variables: residence, region, community-level household heads’ education level, and community-level health insurance.

### 2.11 Operational definitions for community-level variables

Since they were neither observable nor recorded during the survey, all community-level components were computed based on their aggregated values. Each component was calculated according to the value of the corresponding individual variable, following a similar process to that described in previous studies. In this study, a group of homes that shared the primary sample unit or cluster in the dataset was considered a community-level factor. To generate community-level variables, individual-level components were combined. The community variables included region, place of residence, community health insurance status [percentage of households participating in community-based health insurance (CBHI)], community education percentage of household heads with primary or post-primary education, and community wealth (percentage of household heads in the community classified as poor). Continuous community-level variables were further categorized as low or high based on the mean/median value in order to facilitate interpretation of the results (34–36).

#### 2.11.1 Community-level household heads’ educational status

The sum of the educational levels of household heads was based on the typical distributions of education levels in the community. The ratio of household heads in the community with secondary education or higher was classified as low if it was below the median value and high if it was above the median value. The median value was 0.134.

#### 2.11.2 Community-level household wealth status

The same process was employed to obtain the community-level household wealth status variable from each household’s wealth index. In a specific community, the community-level household wealth status was considered high if the ratio of the households from the two quintiles with the lowest levels of wealth was 34.45–100% and low if it was 0–34.44%. The median value was 34.45.

#### 2.11.3 Community-level household health insurance enrollment (CBHI)

The same process was employed to generate the community-level household health insurance enrollment variable from each household’s level of involvement in a community-based health insurance program. It was classified as low if household heads participated in CBHI in the community at a rate of 0–7.7% and high if they did so at a rate of 7.8–100%. The median value was 0.077.

## 3 Results

### 3.1 Sociodemographic characteristics of the study participants

A total of 8,570 weighted households in Ethiopia were included in this study. Among the study participants, 6,675 (77.89%) household heads were male, with an estimated age range of 30–39 years (2,187, 25.53%). More than half of the households (5,455, 63.66%) had up to five household members, and nearly half of the households (4,344, 50.68%) did not have children under the age of five. In terms of wealth status and education levels, approximately 2,095 (24.45%) household members were classified in the richest category, and 4,040 (47.15%) had no formal education. Approximately three-fourths (6,161, 71.90%) of the participants did not enroll in the CBHI scheme. Regarding community-level factors, approximately 5,929 (69.19%) households were from rural areas and 3,171 (37.01%) were from the Oromia region. In terms of the community-level education status and CBHI enrollment, half of the study participants (4,271, 50.16%) scored high in education status, while an equal number of participants (4,298, 50.16%) scored low (Table 1).

**Table 1:** Sociodemographic characteristics of study participants receiving cash or food from the productive safety net program among household members in Ethiopia, (*n* = 8,570).

Variables	Receiving cash or food from the PSNP		Total, *n* (%)
	No, *n* (%)	Yes, *n* (%)	
Sex of the household head			
Male	5,866 (87.88)	809 (12.12)	6,675 (77.89)
Female	1,543 (81.44)	352 (18.56)	1,895 (22.11)
Age of the household head			
15–29	1,539 (92.37)	127 (7.63)	1,666 (19.44)
30–39	1,924 (87.96)	263 (12.04)	2,187 (25.53)
40–49	1,448 (83.72)	281 (16.28)	1,729 (20.18)
50–59	1,004 (81.52)	228 (18.48)	1,232 (14.37)
= > 60	1,494 (85.12)	261 (14.88)	1,755 (20.48)
Number of household members			
1–5	4,795 (87.90)	660 (12.10)	5,455 (63.66)
= > 6	2,613 (83.93)	500 (16.07)	3,114 (36.34)
Enrolling in community health insurance			
No	5,494 (89.16)	668 (10.84)	6,161 (71.90)
Yes	1,915 (79.53)	493 (20.47)	2,408 (28.10)
Education status			
No formal education	3,354 (83.01)	686 (16.99)	4,040 (47.15)
Primary	2,677 (87.50)	382 (12.50)	3,059 (35.70)
Secondary	804 (92.49)	65 (7.51)	869 (10.14)
Higher	574 (95.54)	27 (4.46)	601 (7.01)
Wealth status of the household			
Poorest	1,11 (74.95)	37 (25.05)	1,48 (17.35)
Poorer	1,34 (83.08)	27 (16.92)	1,61 (18.84)
Middle	1,44 (87.11)	21 (12.89)	1,65 (19.29)
Richer	1,54 (89.49)	18 (10.81)	1,72 (20.07)
Richest	1,97 (94.21)	12 (5.79)	2,09 (24.45)
Presence of children under the age of five			
No	3,819 (87.920)	525 (12.08)	4,344 (50.68)
Yes	3,590 (84.95)	636 (15.05)	4,226 (49.32)
Type of residence			
Urban	2,401 (90.92)	240 (9.08)	2,641 (30.81)
Rural	5,008 (84.47)	921 (15.53)	5,929 (69.19)
Regions			
Tigray	472 (81.16)	109 (18.84)	581 (6.78)
Afar	41 (47.35)	46 (52.65)	87 (1.02)
Amhara	1,864 (89.94)	208 (10.06)	2,072 (24.18)
Oromia	2,781 (87.69)	390 (12.31)	3,171 (37.01)
Somali	317 (76.41)	98 (23.59)	415 (4.84)
Benishangul-Gumuz	90 (97.47)	2 (2.53)	93 (1.08)
SNNP	1,393 (83.84)	268 (16.16)	1,661 (19.38)
Gambela	33 (93.69)	2 (6.31)	35 (0.41)
Harari	21 (83.56)	4 (16.44)	25 (0.29)
Addis Ababa	352 (94.23)	22 (5.77)	374 (4.36)
Dire Dawa	45 (81.93)	10 (18.07)	55 (0.64)
Community-level health insurance coverage			
Low	2,582(88.47)	336(11.53)	2,918(34.05)
High	4,827(85.42)	824(14.58)	5,651(65.95)
Community-level poverty			
Low	3,555(83.23)	716(16.77)	4,271(49.84)
High	3,854(89.66)	444(10.34)	4,298(50.16)
Community-level education status			
Low	3,555(83.23)	716(16.77)	4,271(50.16)
High	3,854(89.66)	444(10.34)	4,298(49.84)

### 3.2 Utilization of Productive safety net program among households in Ethiopia by regions

**Figure 1:**
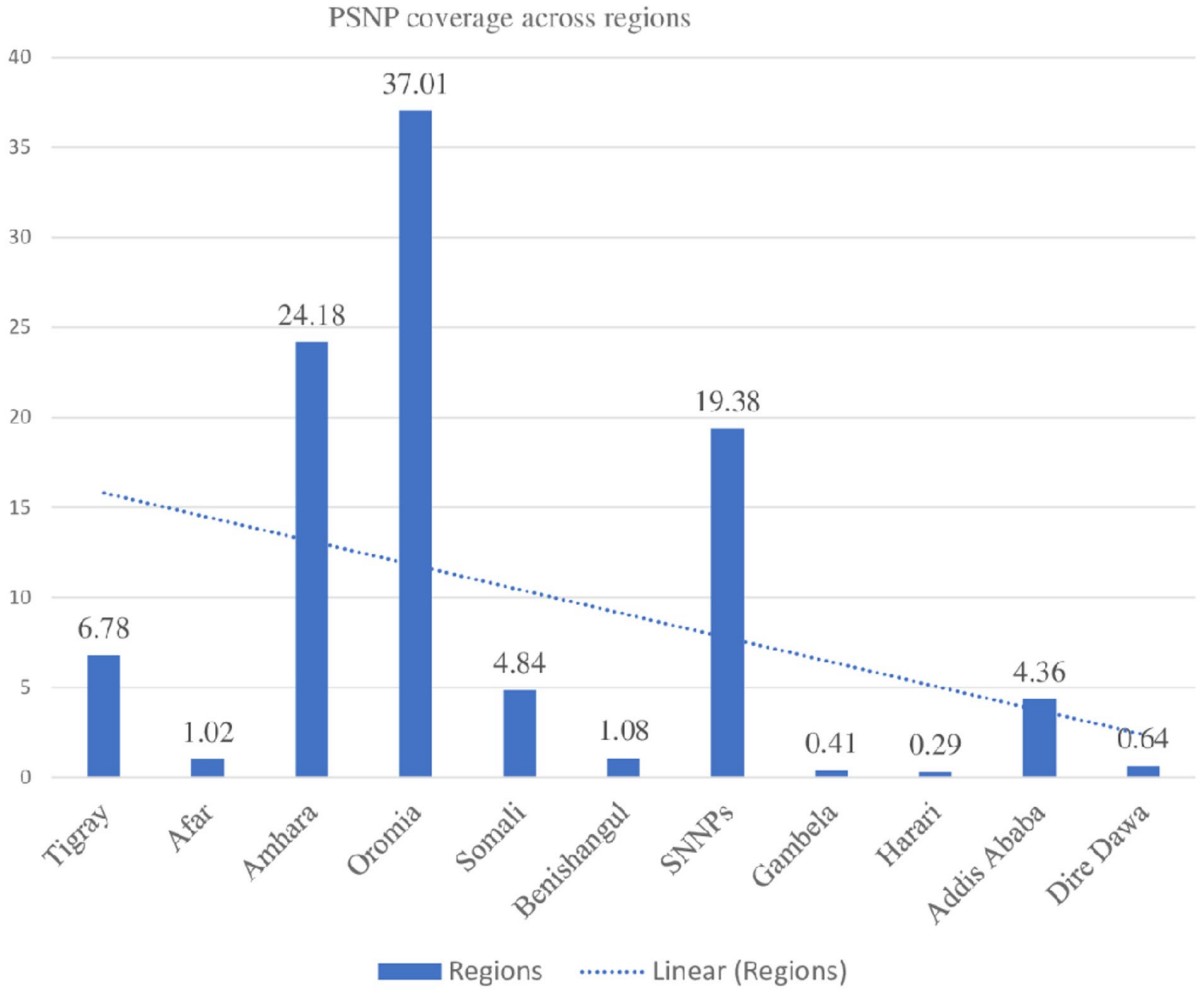
The weighted productive safety net program implementation coverage by regions among households in Ethiopia.

According to the following figure data, the highest proportion of the PSNP was observed in the Oromia region at 37%, and the lowest proportion was observed in the Harari region at 0.25%. However, regions that are highly impoverished, such as Afar, Harari, Gambella, and Somali, showed less than 1% of PSNP coverage (Figure 1).

### 3.3 Spatial distribution of PSNP coverage

**Figure 2:**
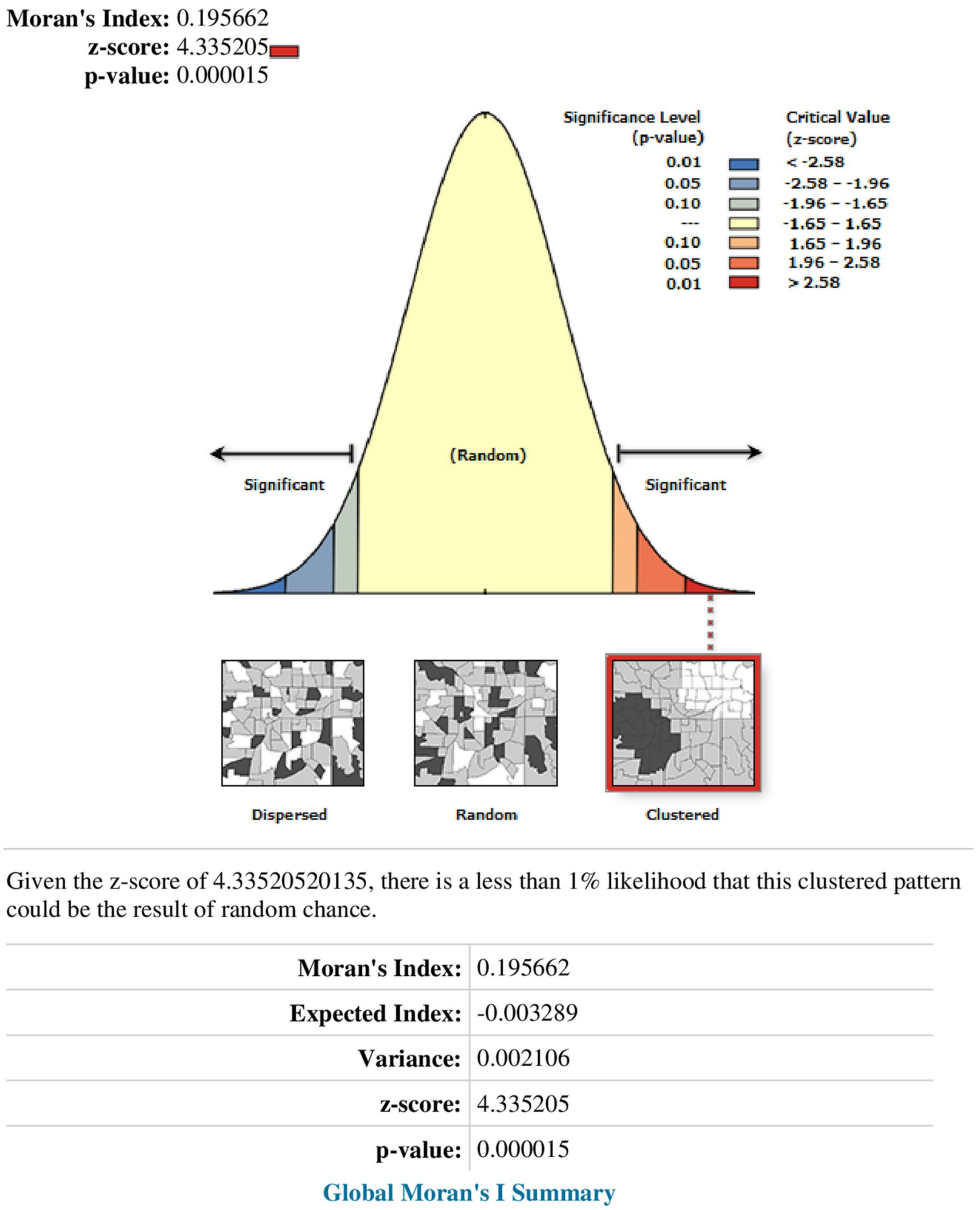
Spatial autocorrelation report analysis of PSNP coverage in Ethiopia.

According to this study, Ethiopia exhibited a spatially clustered pattern of PSNP coverage, as indicated by a Global Moran’s I value of 0.1957 (*p* < 0.0001). There was a concentration of PSNP coverage with high rates observed across the research area. Keys providing information were automatically generated on the right and left sides of each panel. With a Z-score of 4.34, the likelihood of this clustered pattern occurring by chance is less than 1%. The vivid red and yellow coloring in the terminal tails indicates a higher level of importance (Figure 2).

**Figure 3:**
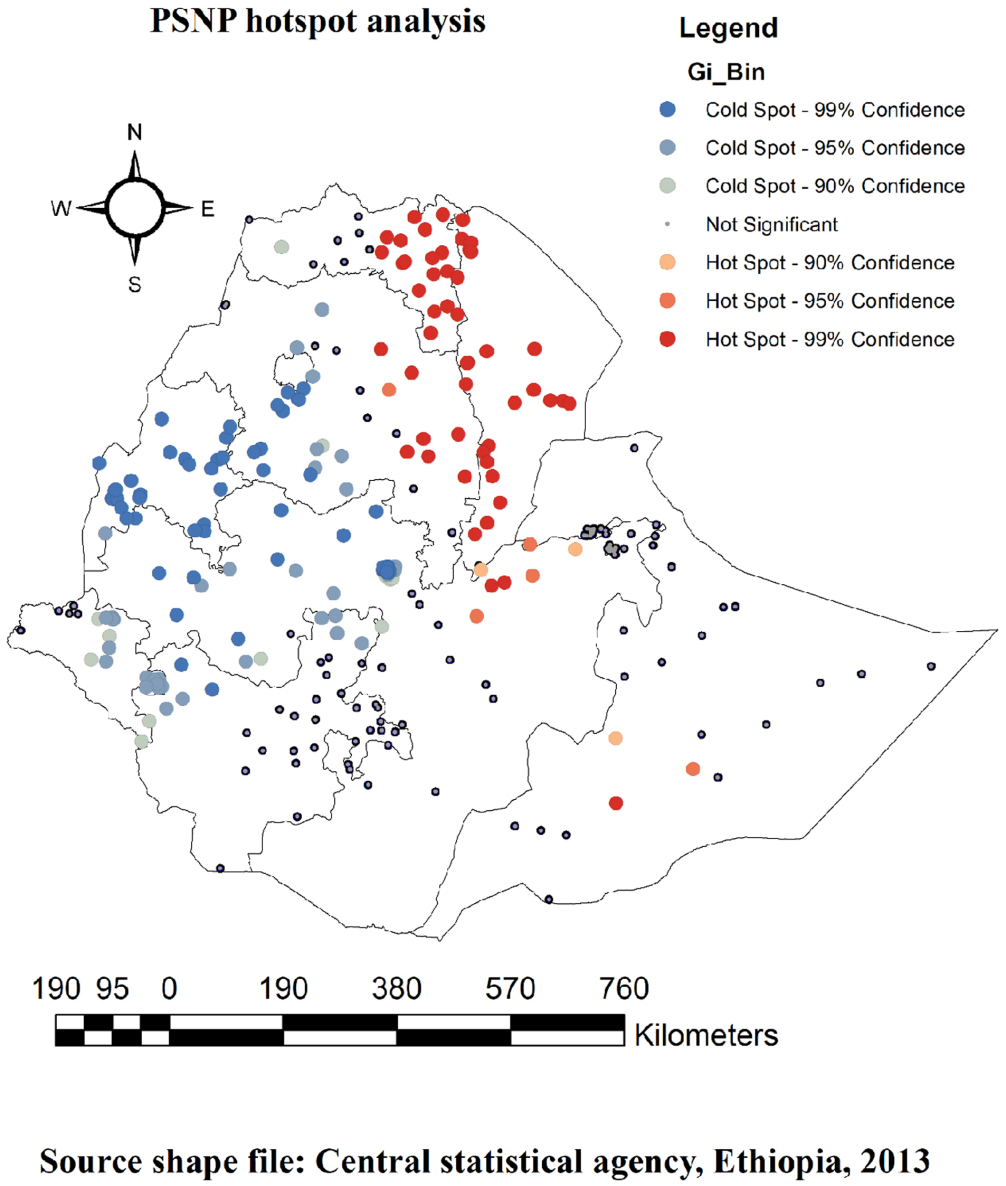
The spatial distribution of PSNP implementation in Ethiopia.

At the regional level, the spatial clustering of PSNP coverage was identified. Out of the weighted 8,570 households surveyed in 2019, only 1,161 (13.54%) had PSNP coverage. The regions of Afar, Dire Dawa, and half of Tigray had the highest rates of PSNP coverage, while the regions of Harari, Somali, most of Oromia, half of Amhara, and Gambella had the lowest rates of PSNP coverage (Figure 3).

### 3.4 Spatial interpolation of PSNP in Ethiopia

**Figure 4:**
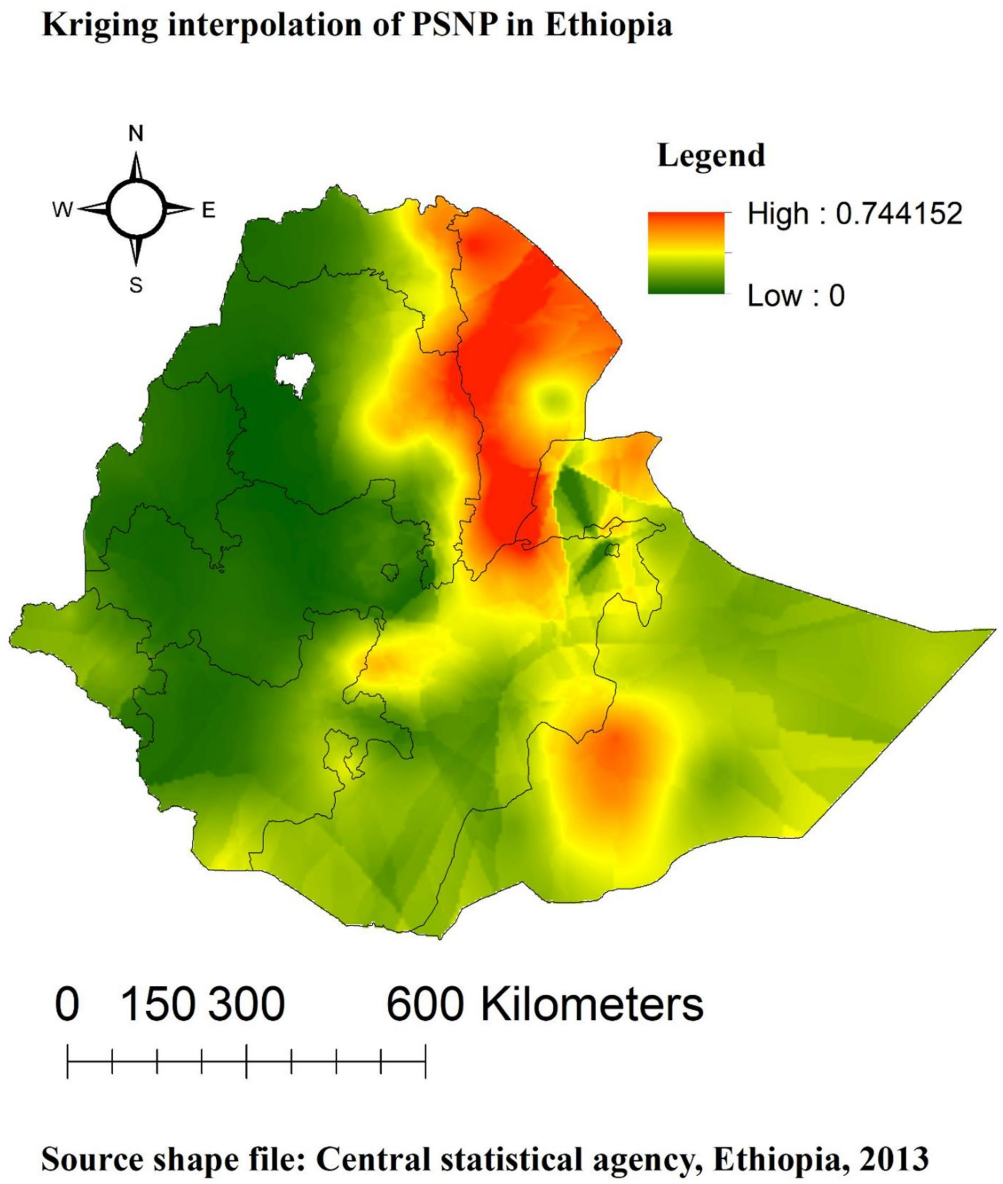
Interpolation predictions of PSNP coverage in Ethiopian across regions.

The PSNP was found to be present in various parts of Ethiopia, practically spanning all of the Afar, eastern Amhara, and Tigray regions, as well as the Dire Dawa region. The utilization of the Kriging interpolation approach resulted in high coverage predictions for the PSNP. Conversely, the remaining regions had low projected PSNP coverage (Figure 4).

### 3.5 Spatial SaTScan analysis of PSNP coverage (Bernoulli-based model)

Both primary and secondary clusters of PSNP coverage were identified as the most likely clusters. The spatial scan statistics analysis of EDHS 2019 revealed a total of 166 significant clusters, consisting of various levels of clusters. Specifically, there were 79 primary clusters, 50 secondary clusters, 3 tertiary clusters, 6 quaternary clusters, 2 quinary clusters, 4 senary clusters, 3 septenary clusters, 15 octonary clusters, 2 nonary clusters, and 2 denary clusters.

The primary cluster, located at 12.346083° N, 41.005800° E with a radius of 332.83 km, was found in the Afar, Dire Dawa, Eastern Amhara, and Tigray regions. It had a relative risk of 3.32, an LLR of 314.60, and a *p*-value of 0.0001. It consisted of a population of 2,248, with 748 cases. Within the cluster, the likelihood of PSNP implementation coverage was 3.32 times higher compared to areas outside the cluster window. The most statistically significant PSNP coverage spatial windows are indicated by vivid green circles.

The secondary cluster, identified as the most likely cluster by SaTScan, was physically separate from the primary cluster. It was located in Ethiopia’s central Oromia region at 12.346083° N, 41.005800° E with a radius of 302.62 km, and it had a population of 1,370, including 582 cases. The cluster had a relative risk of 3.53, an LLR of 312.61, and a *p*-value of 0.0001. PSNP coverage of households within the secondary cluster was 3.97 times more likely than of those outside the cluster window.

**Figure 5:**
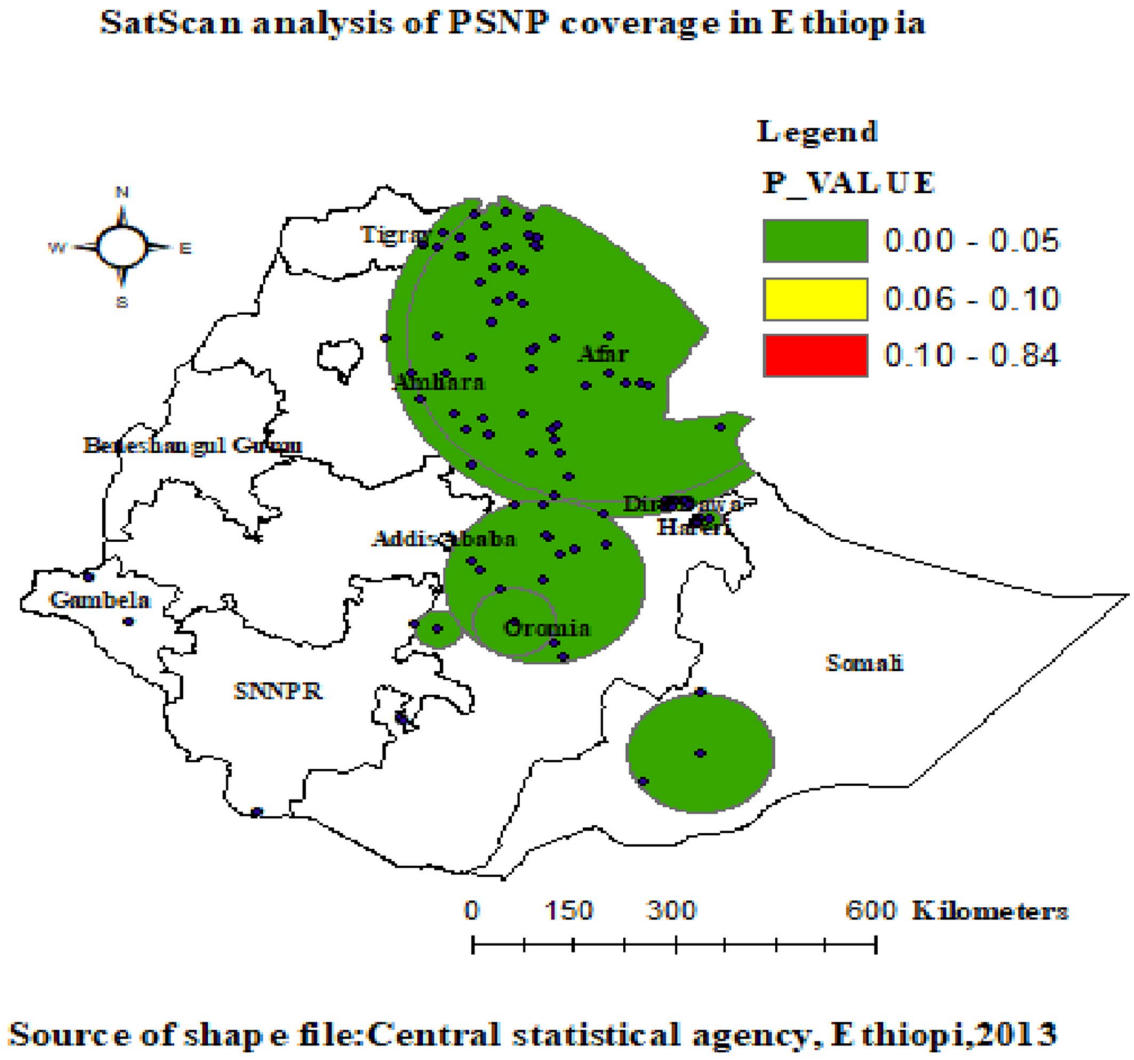
Primary and secondary clusters of PSNP coverage among households across regions in Ethiopia.

Additionally, the analysis showed that the third cluster, primarily found in the eastern and southern parts of Somalia at (5.479641 N, 42.196835 E), had a radius of 108.85 km. This cluster had a relative risk of 3.97, with 86 cases and 56 individuals in the cluster. It had an LLR of 50.19 and a *p*-value of 0.0001. The study population inside this cluster had a 3.97 times greater chance of accessing the PSNP service compared to the population outside the cluster window. For more detailed information, please refer to Table 2 and Figure 5.

**Table 2:** Significant spatial clusters with high-rate productive safety net program coverage among household members in Ethiopia.

Cluster	Enumeration areas (Cluster detected)	Coordinates (radius)	Population	Cases	RR	LLR	*p*-value
1	34, 30, 45, 31, 33, 26, 32, 29, 46, 44, 19, 24, 18, 36, 20, 47, 64, 48, 38, 25, 27, 49, 37, 62, 5, 23, 35, 3, 50, 17, 126, 63, 68, 39,61, 66, 78, 14, 2, 43, 16, 65, 15, 51, 11, 10, 58, 13, 60, 40, 12, 67, 281, 282, 283, 284, 289, 297, 285, 291, 290, 288, 286, 287, 292,293, 298, 294, 296, 295, 7, 301, 305, 302, 304, 303, 42, 299, 127,	(12.346083 N, 41.005800 E) / 332.83 km	2,248	784	3.32	314.60	0.0001
2	34, 30, 45, 31, 33, 26, 32, 29, 46, 44, 19, 24, 18, 36, 20, 47, 64, 48, 38, 25, 27, 49, 37, 62, 5, 23, 35, 3, 50, 17, 126, 63, 68, 39,61, 66, 78, 14, 2, 43, 16, 65, 15, 51, 11, 10, 58, 13, 60, 40	(12.346083 N, 41.005800 E) / 302.62 km	1,370	582	3.53	312.61	0.0001
3	142, 141, 136	(5.479641 N, 42.196835 E) / 108.85 km	86	56	3.97	50.19	0.0001
4	107, 254, 255, 249, 248, 250	(9.312848 N, 42.343386 E) / 18.39 km	174	86	3.04	49.92	0.0001
5	116, 203	(7.531183 N, 38.662596 E) / 34.42 km	56	41	4.43	43.52	0.0001
6	299, 300, 301, 298	(9.592512 N, 42.062390 E) / 8.38 km	112	60	3.26	39.64	0.0001
7	304, 305, 303	(9.514266 N, 41.770584 E) / 7.04 km	83	45	3.28	30.18	0.0001
8	102, 105, 104, 88, 28, 41, 103, 90, 101, 106, 110, 42, 111, 69, 127	(8.313592 N, 40.103390 E) / 149.80 km	419	128	1.88	24.97	0.0001
9	172, 188	(6.066850 N, 38.154184 E) / 8.53 km	60	31	3.10	19.09	0.0001
10	103,104	(7.648661 N, 39.688764 E) / 61.78 km	57	24	2.52	10.06	0.013
11	217	(8.319097 N, 33.925039 E) / 0 km	17	10	3.50	7.57	0.095
12	214	(7.603002 N, 34.494092 E) / 0 km	30	12	2.38	4.49	0.778
13	193	(4.495034 N, 36.230625 E) / 0 km	27	11	2.42	40.70	0.945

### 3.6 Random parameters and model comparison

This study employed multilevel logistic regression to fit the model. Therefore, four fitted models were used to demonstrate the impact of fixed and random parameters, namely the null model, model I, model II, and model III. The null model revealed a significant variance in the probability of enrollment in the PSNP in Ethiopia (community-level variance = 5.29, *p* < 0.001). The intra-cluster correlation coefficient (ICC) in the empty model indicated that community-level differences accounted for 61.67% of the variations in households’ reception of cash and food from the PSNP. This finding suggests that the majority of the overall variability in PSNP utilization is attributed to variations between clusters, with individual differences accounting for the remaining 38.33%. Moreover, the MOR was 8.98 (6.57, 11.38), which implies that as household members transition from low to high reception of cash and food in the PSNP utilization area, the likelihood of PSNP utilization increases by 8.98 times. The population attributable fraction (PCV) indicates the proportion of national variations explained by both community-level and individual-level variables in the empty model. In this case, the combined individual- and community-level model (model III) was the most suitable considering the nature of the data, as it exhibited the lowest deviation value compared to the other models (Table 3).

**Table 3:** Individual and community-level factors associated with receiving cash or food from the productive safety net program among household members in Ethiopia, (*n* = 8,570).

Independent variables	Null model	Model I	Model II	Model III
		AOR [95% CI]	AOR [95% CI]	AOR [95% CI]
*Sex of the household head*				
Male		1		1
Female		1.54 (1.29,1.85)		1.49 (1.24,1.78)
*Age of the household head ^$^*		1.02 (1.01,1.02)		1.02 (1.01,1.02) *
*Number of household members^$^*		1.05 (1.02,1.09)		1.05 (1.021.10) *
*Education status*				
No formal education		1		1
Primary		0.86 (0.71,1.04)		0.87 (0.72,1.06)
Secondary		0.87 (0.63,1.21)		0.87 (0.63,1.19)
Higher		0.45 (0.28,0.72)		0.45 (0.28,0.71) *
*Wealth status of the household*				
Poorest		1		1
Poorer		0.84 (0.65,1.07)		0.94 (0.73,1.21)
Middle		0.61 (0.46,0.82)		0.72 (0.54,0.97) *
Richer		0.38 (0.27,0.53)		0.46 (0.33,0.64) *
Richest		0.23 (0.16,0.35)		0.26 (0.17,0.41) *
*Health insurance enrolment*				
No		1		1
Yes		3.06 (2.47,3.81)		3.21 (2.58,4.01) *
*Presence of children under the age of five*				
No		1		1
Yes		1.16 (0.96,1.39)		1.15 (0.95,1.39)
*Type of residence*				
Urban			1	1
Rural			1.90 (0.96,3.75)	1.11 (0.55,2.23)
*Regions*				
Tigray			1	1
Afar			17.44 (6.30,48.23)	21.06 (7.57,58.66) *
Amhara			0.22 (0.09,0.54)	0.17 (0.07,0.44) *
Oromia			0.42 (0.17,1.02)	0.48 (0.19,1.18)
Somali			1.93 (0.69,5.39)	1.81 (0.64,5.08)
Benishangul-Gumuz			0.06 (0.02,0.19)	0.07 (0.020.24) *
SNNPs			0.65 (0.27,1.61)	0.75 (0.30,1.85)
Gambela			0.50 (0.17,1.43)	0.63 (0.22,1.83)
Harari			1.64 (0.59,4.59)	2.53 (0.90,7.09)
Addis Ababa			1.34 (0.44,4.15)	2.17 (0.69,6.76)
Dire Dawa			3.30 (1.18,9.19)	5.29 (1.89,14.81) *
*Community-level health insurance coverage*				
Low			1	
High			3.65 (2.22,5.99)	2.49 (1.51,4.11) *
Community-level education				
Low			1	1
High			0.94 (0.54,1.63)	1.13 (0.65,1.97)
*Community-level poverty*				
Low			1	1
High			4.22(2.39,7.41)	2.68(1.51,4.79) *
Random parameters and model comparison				
Community-level variance	5.29 (4.15,6.76)	4.49 (3.49,5.76)	2.33 (1.81,3.03)	2.28 (1.76, 2.96)
ICC (%)	61.67	57.7	41.52	40.95
MOR (95% CI)	8.98 (6.57,11.38)	7.55 (5.64,9.45)	4.30 (3.49,5.11)	4.22 (3.43,5.02)
PCV (%)	Reference	0.18	1.30	1.32
Log-likelihood (LLR)	−2781.64	−2632.96	−2699.47	−2562.43
DIC (−2LLR)	5,563.28	5,265.92	5,398.94	5,124.86
AIC	5567.28	5293.91	5430.94	5180.87
BIC	5581.38	5392.67	5543.79	5378.37

$ and * indicate continuous variables and significance level at a *p*-value of < 0.05 and continuous variables in the regression, respectively.

### 3.7 Factors associated with receiving cash and food from PSNP in Ethiopia

Determinants such as age, sex, household size, education status, wealth status, community-based health insurance enrollment, community-level poverty, community-level health insurance coverage, family size, and regions were found to be statistically significant in the multilevel multivariable logistic regression model for PSNP utilization in Ethiopia.

As the age of the household head and the family size increased by one unit, the odds of PSNP utilization also increased (adjusted odds ratio (AOR) = 1.02, 95% CI 1.01–1.02) and (AOR = 1.05, 95% CI 1.02–1.10), respectively. Household members with higher education attainment had a lower odds ratio of being involved in the PSNP (AOR = 0.45, 95% CI 0.28–0.71). Similarly, participants from the middle (AOR = 0.72, 95% CI 0.54–0.97), richer (AOR = 0.46, 95% CI 0.33–0.64), and richest (AOR = 0.26, 95% CI 0.17–0.41) wealth classes had lower odds ratios of accessing the PSNP in Ethiopia compared to other wealthy classes. However, households enrolled in community health insurance had a higher likelihood of utilizing the PSNP compared to those without enrollment (AOR = 3.21, 95% CI 2.58–4.01). Among community-level variables, three variables—region, community-level education status, and community-level community-based health insurance coverage—showed statistically significant associations with the outcome variable. The odds of PSNP utilization decreased by 0.83 in the Amhara region (AOR = 0.17, 95% CI 0.07–0.44) and 0.93 in the Benishangul-Gumuz region (AOR = 0.07, 95% CI 0.02–0.24). The highest statistical association was found in the Afar region (AOR = 21.06, 95% CI 7.57–58.66) and the Dire Dawa regional administrative city (AOR = 5.29, 95% CI 1.89–14.81). Household members belonging to communities with high levels of community-based health insurance coverage and communities with higher poverty levels had a positive statistically significant association with PSNP utilization in Ethiopia, with AOR of 2.49 (95% CI: 1.51–4.11) and 2.68 (95% CI 1.51–4.79), respectively (Table 3).

## 4 Discussion

This study aimed to explore the spatial clustering and potential determinants that may have positive or negative effects on the implementation coverage of cash and food reception from the PSNP in Ethiopia. The study utilized data from the EDHS. It revealed that the coverage of cash and food reception from the PSNP among households in Ethiopia is 13.54% (95% CI, 12.84–14.29).

In the final model regression, households with female heads showed a higher probability of joining the PSNP compared to households with male heads. This result indicates the socioeconomic impact of female household heads on the entire family and that female heads are a key to household food security (37). This finding shows that women, girls, and children are among the most affected, highlighting the role of the head of the household for these groups (38, 39). In Ethiopia, as in other African nations, most household headship and activities are dominated by male decision-making (40). Funding is entirely controlled by men. Therefore, uplifting women’s education, attitude, and economic and social participation is critical to ensuring family food security in Ethiopia.

The second paramount variables were the age of the household heads and family size. The study at hand discovered that, as the age of the household head and family size increased by a unit, the probability odds ratio of joining and being included by the PSNP also increased. Previous studies have obtained similar findings in various settings (41–43). This phenomenon may be due to the fact that, as household heads’ age increases, they find it difficult to feed themselves and their families; however, their expenses depend on the number of family members and various social-and health-related challenges, so their ability to ensure food security may decrease over time (44). All these conditions force them to join the PSNP. The research results obtained in this study indicate that an increase in age and family size has a negative impact on food security and the economy.

Two important variables showed a statistically significant association with the PNSP. Those household heads who have completed their college and university education and belong to the middle, richer, or richest households had a lower likelihood of becoming a member of the PSNP compared to household heads belonging to the poorest households. This difference might be due to their higher education and better wealth status. Household heads with higher levels of education may have better lifestyles, better incomes, and may be less inclined to join the PSNP than those with no formal education. This evidence is supported by several studies conducted at various places (45–47). Similarly, household heads that are in a better economic position will not join the PSNP because they will have the ability to meet the food security of their families to some extent compared to poor household heads (43). Since the PSNP is for the poor and those whose food security is not ensured, there is a low probability that households with better incomes will have the desire to join the PSNP (48–50). Furthermore, the family units nominated for the PSNP are recruited separately by the lowest-level administrative and district leaders at various levels, so even if these households with better income request for aid, they will not be accepted by their leaders.

This study revealed that, in the factor analyses of both the individual and community levels, the community-based health insurance scheme showed a positive statistically significant association with the PSNP. This is possible because the objectives of these two social programs are almost the same. The main purpose of the community-based health insurance scheme is to save and protect people from unnecessary expenses caused by medical expenses and catastrophes. Therefore, people who join the health insurance scheme are more likely to receive information and request questions and clarifications on the PSNP from the people and other concerned bodies, or the information might be disclosed to them by the concerned bodies at various contacts and meetings about the policy of the country. Consequently, they are better than the rest of the households in terms of understanding and demonstrating willingness to accept and utilize the PSNP. Therefore, since these two programs function closely with each other, if a relevant party sends a message to health insurance beneficiaries to join the PNSP and use it properly, access to the PNSP will be more efficient and effective. Food insecurity is an important public health issue. It affects significant portions of those households that face hunger and has a variety of detrimental health effects and vice versa (51, 52), i.e., families with food insecurity frequently put off getting required medical care and prescriptions (53).

Another significant factor is region. In this study, two regions, namely Amhara and Benishangul-Gumuz, showed a lower likelihood of becoming a member of the PSNP compared to the Tigray region. Although the Ethiopian government started the PSNP in the Tigray, Amhara, Oromia, and SNNPs regions, it has currently expanded its reach to include areas such as Afar, Somalia, and Dire Dawa, mostly concentrating on developing and infertile areas that are experiencing drought (54, 55). This may result in a decrease in the Amhara region’s prior percentage, and although the Amhara region is classified as one of Ethiopia’s more developed regions, the implementation of the PSNP could suffer as a result. However, the other two regions of the country, namely the Afar region and Dire Dawa regional city, have shown a higher likelihood of enrolling in the PNSP compared to the Tigray region. It is possible that the Afar region is composed of deserts, high drought areas, poorly developed infrastructure, widely dispersed inhabitants, and pastoral communities that occasionally experience chronic poverty and food insecurity (54, 56). Millions of Ethiopians have faced hunger over the years, especially in areas such as Afar, due to drought and an inability to produce adequate food. According to recent reports, the Ethiopian government and the World Food Program will both invest in regional and woreda capacity building, particularly in pastoral and agro-pastoral areas such as the Afar region, and continue to support the government’s implementation of the program. With households taking part in the PSNP, the government will continue its Household Asset Building Program. Numerous data from the Ethiopian government, the World Health Organization, the World Bank, and other organizations show that urban food insecurities vary in the big cities, such as Dire Dawa and Addis Ababa, of developing countries such as Ethiopia (55). The Ethiopian government created the Urban Food Security Strategy in 2015 through safety net initiatives to address this issue. In addition to addressing the rising levels of vulnerability, inequality, and poverty, the strategy’s goal was to reduce urban food insecurity (57, 58). The PSNP in Dire Dawa city may be improved as a result of these governmental measures.

## 5 Conclusion

The implementation rate of PSNP in Ethiopia was found to be low. Age and sex of the household heads, family size, level of education and household wealth, community-based health insurance enrollment, and region were found to be statistically significant with the PSNP in Ethiopia. The country is still in extensive need of cash and food safety net programs, and it needs comprehensive implementation strategies to enhance it. The Tigray, Afar, eastern Amhara, and Dire Dawa regions had the greatest proportion of PSNP coverage, while Benishangul-Gumuz, Addis Ababa, Somalia, Harari, and the majority of Oromia had the lowest proportion of PSNP coverage. Owing to PSNP coverage varying significantly between regions in Ethiopia, programmers must develop consistent financial and technical assistance as well as raise awareness for these areas. Since the PSNP was only launched by the Ethiopian government in four regions, developing regions and major cities require extensive rehabilitation and attention to become self-sufficient. Similarly, encouraging women empowerment, community-based awareness creation, and coordination with regional states is advisable.

### 5.1 Strategies for enhancing implementation and coverage of the PSNP in Ethiopia

Based on our findings, the following strategies and implementation policies can be considered to improve PSNP coverage across regions and households in Ethiopia:

Targeted Outreach: Develop targeted outreach programs to reach households in regions with low PSNP coverage, such as Benishangul-Gumuz, Addis Ababa, Somalia, Harari, and most parts of Oromia. These programs should focus on raising awareness about the program, its benefits, and eligibility criteria.

Enhanced Accessibility: Improve accessibility to the PSNP by establishing more distribution centers in areas with low coverage. This can include setting up additional distribution points and mobile distribution units or collaborating with local community centers to facilitate easier access for eligible households.

Strengthened Regional Collaboration: Foster collaboration between regions with high PSNP coverage, such as Tigray, Afar, Eastern Amhara, and Dire Dawa, and those with low PSNP coverage. This collaboration can involve sharing best practices, lessons learned, and strategies for effective implementation to improve coverage in regions that are lagging behind.

Customized Approaches: Recognize the unique challenges and needs of different regions and tailor implementation strategies accordingly. The following factors can be taken into consideration: cultural sensitivities, local economic conditions, and specific barriers to program participation in each region.

Capacity Building: Invest in capacity building initiatives for local authorities and stakeholders involved in PSNP implementation. The initiatives can include training programs for enhancing their knowledge and skills in program management, monitoring, and evaluation. Strengthening the capacity of local institutions will contribute to more effective implementation and improved PSNP coverage.

Improved Data Collection and Monitoring: Enhance data collection and monitoring systems to ensure accurate and up-to-date information on PSNP coverage. This will enable timely identification of gaps and challenges, allowing for targeted interventions to address them.

Collaboration with Stakeholders: Collaborate with relevant stakeholders, including local communities, civil society organizations, and development partners, to leverage their expertise, resources, and networks. Engaging with these stakeholders will foster ownership and support for the PSNP, which will lead to improved implementation and coverage.

Continuous Evaluation and Adaptation: Regularly evaluate the effectiveness of implemented strategies and policies and make necessary adaptations based on the findings. This iterative approach will enable learning from experience and continuous improvement of PSNP implementation across regions and households in Ethiopia. By implementing these strategies and policies, Ethiopia can work toward enhancing the coverage and effectiveness of the PSNP, which will address the extensive need for a cash and food safety net program in the country.

## Data availability statement

The raw data supporting the conclusions of this article will be made available by the authors, without undue reservation.

## Ethics statement

Ethical approval was not required for the study involving humans in accordance with the local legislation and institutional requirements. The studies were conducted in accordance with the local legislation and institutional requirements. Written informed consent to participate in this study was not required from the participants in accordance with the national legislation and the institutional requirements.

## Author contributions

BT: Conceptualization, Formal analysis, Methodology, Software, Writing – original draft, Writing – review & editing. BeM: Data curation, Formal Analysis, Funding acquisition, Investigation, Resources, Visualization, Writing – review & editing. WS: Investigation, Methodology, Resources, Supervision, Validation, Writing – review & editing. BiM: Data curation, Investigation, Project administration, Resources, Supervision, Validation, Visualization, Writing – review & editing.
